# Region- and Compartment-Specific Elevation of Bone Mass in Mice Following *Tsc1* Deletion in 8-kb Dmp1-Cre-Expressing Cells

**DOI:** 10.1007/s00223-026-01532-8

**Published:** 2026-04-27

**Authors:** Iya Ghassib, Anusha Inti, Nushaba Hossain, Thomas Kim, Danielle Moon, Lu Han, Rawan Mohsen, Honghao Zhang, Nicholas Auyeung, Yuji Mishina, Daniela Mendonça, Darnell Kaigler, Teresita Bellido, Fei Liu

**Affiliations:** 1 Department of Biologic and Materials Sciences & Prosthodontics, University of Michigan School of Dentistry, 1011 North University Avenue, Ann Arbor, MI 48109-1078 USA; 2 Department of Periodontics and Oral Medicine, University of Michigan School of Dentistry, Ann Arbor, MI USA; 3 Department of Physiology and Cell Biology, University of Arkansas for Medical Sciences, Little Rock, AR USA; 4 Present Address: Virginia Commonwealth University School of Dentistry, Richmond, VA USA

**Keywords:** mTORC1 signaling, *Tsc1*, Dmp1-Cre, Ai14, Cortical bone, Trabecular bone, Craniofacial

## Abstract

Tuberous sclerosis complex 1 (TSC1) is a negative regulator of mTORC1 signaling, a key pathway in skeletal homeostasis, but its region- and compartment-specific effects on bone remain unclear. Here, we examined the skeletal impact of *Tsc1* deletion using the 8-kb Dmp1-Cre line, which targets mature osteoblasts and osteocytes. Cre activity in Ai14 reporter mice showed comparable recombination in craniofacial, appendicular, and axial bones. Conditional *Tsc1* deletion resulted in greater bone mass at multiple sites, but with marked regional and compartmental variations. In the craniofacial skeleton, the frontal bone showed the most robust elevation in thickness and volume, the parietal bone displayed intermediate elevation, and the mandible exhibited the smallest elevation but reduced bone volume fraction and tissue mineral density, consistent with greater porosity. In femurs, both trabecular and cortical bone mass was augmented. Histomorphometric analysis of femoral trabecular bone revealed increased osteoblast number and surface together with reduced osteoclast parameters. In vertebrae, *Tsc1* deletion produced modest trabecular bone accrual but > 100% expansion of both outer and inner cortical bone, indicating a predominant cortical effect. Comparative analysis showed stronger trabecular bone response in the femur and greater cortical response in the vertebra. p-S6 immunostaining confirmed mTORC1 activation across skeletal sites. Comparison with Osterix-Cre–mediated *Tsc1* deletion showed similar trabecular responses but milder cortical effects in Dmp1-Cre meditated *Tsc1* deletion, suggesting stage-specific regulation within the osteoblast lineage. These findings reveal pronounced regional variation among craniofacial bones and distinct compartmental responses to *Tsc1* deletion, highlighting the role of local bone context in shaping skeletal outcomes of mTORC1 hyperactivation.

**Supplementary Information:**

The online version contains supplementary material available at https://doi.org/10.1007/s00223-026-01532-8.

## Introduction

Tuberous sclerosis (TS) is an autosomal dominant disorder caused by pathogenic variants in *TSC1* or *TSC2*, leading to constitutive activation of mTORC1 [1]. Although best known for hamartomatous lesions in the brain, skin, and kidneys, TS also produces distinctive osseous abnormalities. Sclerotic bone lesions have been reported in both the axial and appendicular skeleton and were recently incorporated as a minor diagnostic criterion [2]. In a radiographic survey of 43 patients, abnormal sclerotic islands in the cranial vault were present in ~ 40%, while cyst-like lesions in the phalanges and wavy periosteal thickening of the metatarsals or metacarpals occurred in ~ 66% [3]. These osseous changes can vary in distribution and severity, underscoring the potential for site- and region-specific skeletal effects of *TSC* gene loss.

Genetic studies demonstrate that the skeletal effects of *Tsc1* loss depend strongly on the targeted stage of the mesenchymal and osteoblast lineage cells. Neural crest-specific deletion (*P0*-Cre) caused craniofacial sclerosis via early postnatal expansion of osteoprogenitors [4]. Mesenchymal stromal/stem cell deletion (*Prx1*-Cre) widened bones and increased mass but reduced length and mineral content, while suppressing osteoclastogenesis [5]. Huang et al. showed that preosteoblast deletion (*Osterix*-Cre) induced hyperproliferation but impaired maturation, yielding woven bone through STAT3/p63/Jagged/Notch activation and Runx2 suppression [6]. Also using *Osterix*-Cre, we found reduced femoral trabecular bone and increased marrow adiposity due to autophagy suppression, Notch1 accumulation, and β-catenin degradation [7]; our data from the same model revealed early cortical and calvarial bone mass elevation [8]. By contrast, osteocyte-targeted deletion with 10-kb *Dmp1*-Cre lowered sclerostin via mTORC1-dependent Sirt1 down-regulation, enhancing osteogenesis despite elevated RANKL and osteoclast formation [9]. Collectively, these models reveal potent, stage-dependent mTORC1 effects on bone mass and quality, yet none have systematically compared multiple skeletal sites and compartments within a single model.

The *Dmp1* promoter has been widely used to target late-stage osteoblasts and osteocytes, with two common Cre-driver constructs: 10-kb and 8-kb *Dmp1*-Cre. The 10-kb version shows strong activity in osteocytes and odontoblasts but also recombines in some osteoblasts and in extra-skeletal tissues, including brain, kidney, and muscle [10–12]. The 8-kb *Dmp1*-Cre, derived from a shorter upstream regulatory segment, was generated to improve osteocyte specificity based on the more restricted pattern of 8-kb *Dmp1*-GFP expression [13, 14]. Structurally, the 8-kb *Dmp1*-Cre includes 8 kb of the 5′ flanking region, the first exon, the first intron, and 17 bp of exon 2 of the murine *Dmp1* gene [14]. Cre mRNA is readily detectable in bone but not in kidney, heart, brain, or skeletal muscle [14], and reporter studies show recombination in osteocytes, some osteoblasts, muscle, and very few bone marrow cells [11]. Given our interest in defining mTORC1 function in the osteocyte lineage and evaluating skeletal effects across different bones, we used 8-kb *Dmp1*-Cre to delete *Tsc1* and systematically assess site- and compartment-specific phenotypes.

## Materials and Methods

### Mice

To characterize the recombination activity of 8 kb Dmp1-Cre, 8-kb Dmp1-Cre (Dmp1-Cre hereafter) mice [14] were crossed with Ai14 reporter mice (ROSA26-tdTomato, red fluorescence) [15] to generate 8 kb Dmp1-Cre mice; Ai14 mice. 8 kb Dmp1-Cre mice were maintained in C57BL/6 background and Ai14 mice were maintained on a mixed 129/C57BL/6 background. One-month-old males and females were used for Cre activity assessment. For conditional deletion of *Tsc1*, there are three experimental groups including 2-month-old *Tsc1* conditional knockout (CKO, *Tsc1*^Flox/Flox^; Dmp1-Cre/+), *Tsc1* conditional heterozygote (CHet, *Tsc1*^Flox/+^; Dmp1-Cre/+), and sex-matched control (CTR, *Tsc1*^Flox/Flox^ or *Tsc1*^Flox/+^ [16]) mice from the same litters, generated by breeding CHet male mice and CTR female mice. The genotypes were determined using reported primers [16] to detect *Tsc1* floxed allele and Cre transgene. The mice containing *Tsc1* floxed alleles and Cre transgene without *Tsc1* wild-type allele were designated as CKO. The mice containing both *Tsc1* floxed allele, *Tsc1* wildtype allele, and Cre transgene were designated as CHet. The mice containing either *Tsc1* floxed alleles or both *Tsc1* floxed and wild-type alleles without Cre transgene were designated as CTR. *Tsc1*^Flox/Flox^ mice were maintained in C57BL/6 background. Two-month-old males and females were used for experiments. All mice were housed under standard conditions at the University of Michigan School of Dentistry. All procedures were approved by the University of Michigan Institutional Animal Care and Use Committee (IACUC). Genotypes were confirmed by PCR analysis of tail DNA as reported.

### Sample Processing and Immunofluorescence

Two-month-old mice were euthanized by CO_2_ inhalation followed by cervical dislocation. Calvaria, mandibles, and femurs were dissected and fixed in 4% paraformaldehyde (PFA) for 4 days, then transferred to 70% ethanol. Calvaria and mandibles were decalcified in 14% EDTA (pH 7.2) for 24 and 21 days, respectively, followed by overnight immersion in 30% sucrose at 4 °C. Samples were embedded in OCT compound (Tissue-Plus O.C.T. Compound, Fisher Healthcare, USA), and sectioned at 10 µm using a cryostat. Sections included the right mandible and calvaria (frontal and parietal bones). Each specimen was sectioned in triplicate and mounted on glass slides. For fluorescent imaging, sections were stained with DAPI. Adjacent sections were counterstained with hematoxylin and xylene to confirm tissue structure and integrity. Imaging was performed with a Zeiss LSM 710 laser scanning confocal microscope. ImageJ software was used for quantification analysis.

### Nano-CT

Bone morphometric analysis was performed using high-resolution cone-beam nano-computed tomography (nano-CT; eXplore Locus SP, GE Healthcare Pre-Clinical Imaging, London, ON, Canada) as described in detail in our recent publication [8]. Calvaria, mandibles, and femurs from 2-month-old male and female mice were fixed in formalin for 3 days and stored in 70% ethanol prior to scanning. In brief, scans were acquired with an 80 kV, 400 μA X-ray source, diamond-coated tungsten target, 0.381-mm aluminum filter, 500-ms exposure, and 3-frame averaging, with 2000 projections over 360° rotation. Reconstruction was performed using Datos.rec v2.6.1.Images were reconstructed at 8 µm (femur and verterbra) or 10 µm (calvaria and mandible) isotropic voxel size, and analyses were performed using MicroView software (version ABA 2.2). The femur analysis was performed with the same protocol as we described previously [17, 18]. The vertebra (the third lumbar) analysis was performed as we described before [19]. Trabecular bone was isolated by outlining the endosteal border and excluding the cortical shell. Cortical measurements were derived from diaphyseal or vertebral slices with no trabecular elements, following the standardized anatomical boundaries used in our prior work. All mineralized tissue was segmented using the auto-Threshold function in MicroView, applied uniformly to all samples without manual adjustment. The analysis of calvariae was performed as we previously reported [20, 21]. In brief, For the ROI of frontal bone was located 1.5 mm anterior to the coronal suture and 1 mm lateral to the midsagittal line; and the ROI of parietal bone was located 1.5 mm posterior to the intersection of the interfrontal and coronal sutures. ROI volume was 0.5 × 0.5 × bone thickness (mm). For mandibular cortical bone, the ROI extended from the distal root of the first molar to the mesial periodontal ligament of the second molar, with the inferior boundary defined by the lower border of the mandible. ROI volume was 0.3 × mean bone height × bone thickness (mm). Bone thickness was calculated as the mean of three equally spaced measurements (initial, middle, and final slices) using ImageJ software. The ROI of calvaria and mandible includes full thickness of bone without separating cortical versus trabecular bones.

### Statistical Analysis

Volumetric bone parameters were compared between three genotypes within each sex using one-way ANOVA. Two-way ANOVA was used to compare percentage increases in frontal, parietal, and mandibular bone parameters between males and females. Percentage increase was calculated as (CKO-CTR)/CTR 100%. Graphs and analyses were performed using GraphPad Prism.

## Results

### 8-kb Dmp1-Cre Activity Characterization Using Ai14 Reporter Mice

**Fig. 1:**
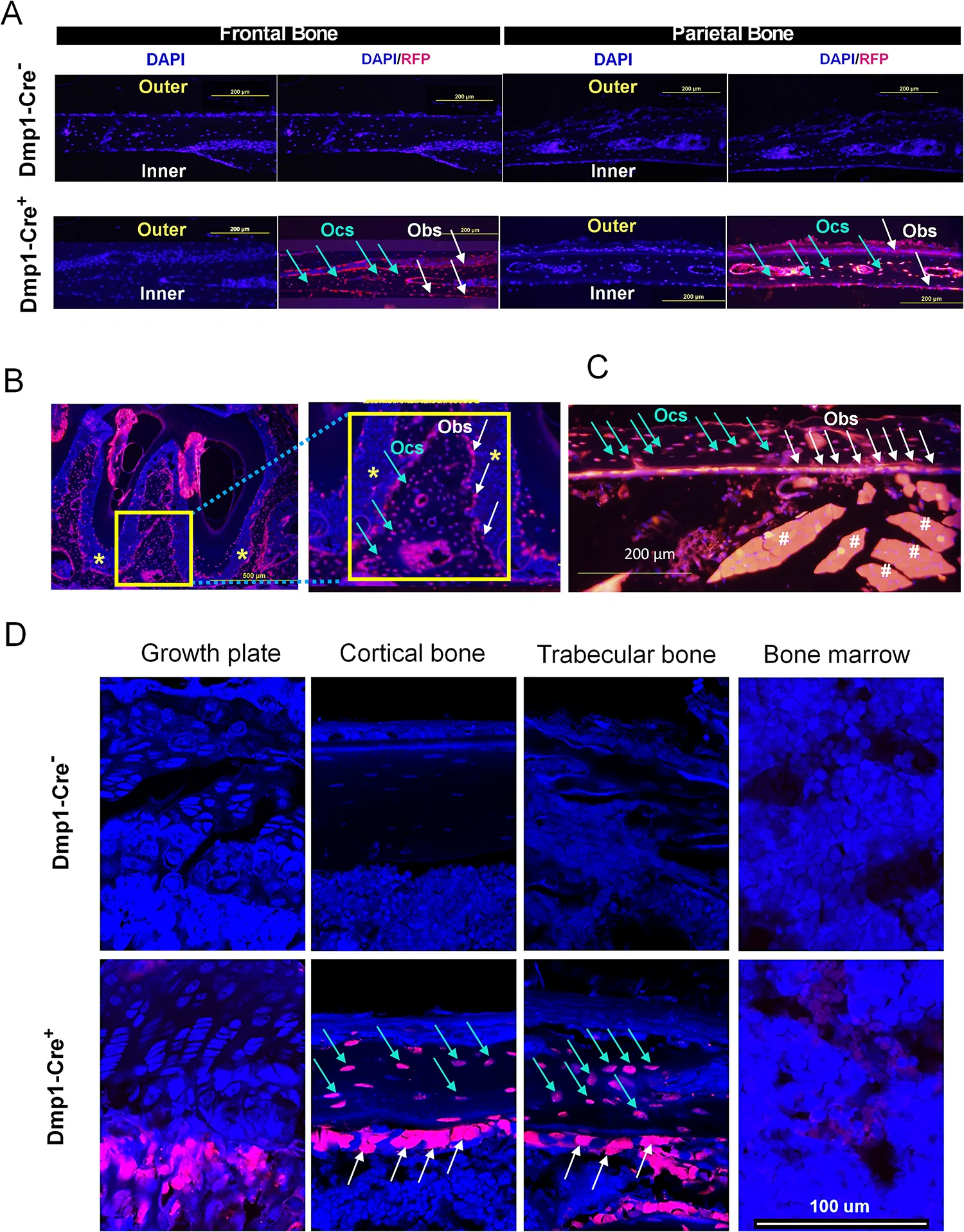
Cre activity in 1-month-old 8-kb Dmp1-Cre;Ai14 reporter mice. Dmp1-Cre mice were crossed with Ai14 (ROSA26-tdTomato) reporter mice to visualize Cre activity (RFP, magenta; nuclei, DAPI, blue). White arrowheads indicate osteoblasts and blue arrows indicate osteocytes throughout all panels. **A** Frontal and parietal bones showing robust labeling of osteoblasts and osteocytes on both the extracranial (outer) and intracranial (inner) surfaces of the calvaria. **B** Alveolar bone between tooth roots with strong labeling in osteoblasts and osteocytes; *indicates periodontal ligament lacking RFP signal. **C** Mandible with extensive labeling of osteoblasts and osteocytes; # indicates RFP-positive muscle adjacent to the mandible. **D** Femur sections: upper panel, Dmp1-Cre^−^ control; lower panel, Dmp1-Cre⁺ sample. From left to right—growth plate, cortical bone, trabecular bone, and bone marrow—showing osteoblast and osteocyte labeling in cortical and trabecular regions, some positive hypertrophic chondrocytes in the growth plate, and minimal labeling in bone marrow. Scale bars: 200 μm (**A**, **C**), 500 μm (**B**), 100 μm (**D**)

To assess the tissue distribution of Cre activity driven by the 8-kb Dmp1 promoter, we crossed Dmp1-Cre mice with Ai14 reporter mice, in which red fluorescence (tdTomato) indicates Cre-mediated recombination. While the activity of this Cre line has been partially characterized—particularly in long bones—its expression pattern in craniofacial regions has not been well defined. In 1-month-old reporter mice, robust tdTomato signals were detected in osteocytes of the frontal and parietal bones, and many osteoblasts on both extracranial and intracranial surfaces were also positive (Fig. 1A). In the alveolar bone between tooth roots, most osteocytes and lining osteoblasts expressed tdTomato, whereas the periodontal ligament showed little to no signal (Fig. 1B). In the mandibular body, the majority of osteocytes and osteoblasts were positive (Fig. 1C). Notably, strong fluorescence was also observed in the muscles attached to the mandible (Fig. 1C). In the femur, consistent with prior reports, most osteocytes and some osteoblasts in both cortical and trabecular bone were positive, while bone marrow cells were largely negative. Additionally, tdTomato expression was observed in some hypertrophic chondrocytes (Fig. 1D).

### Deletion of *Tsc1* by 8-kb Dmp1-Cre Differentially Affects Craniofacial Bone Acquisition

**Fig. 2:**
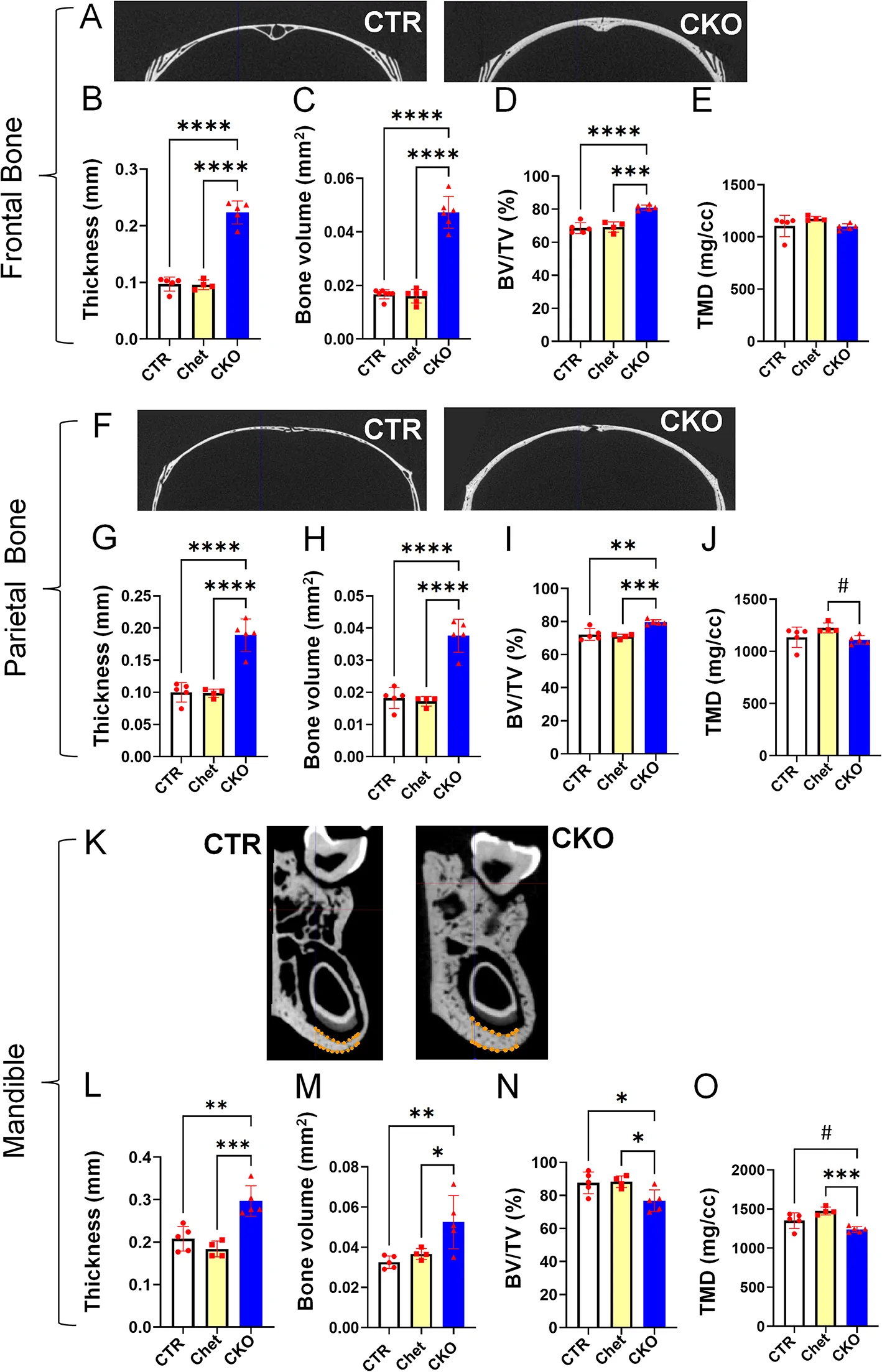
*Tsc1* deletion by 8-kb Dmp1-Cre leads to greater craniofacial bone mass in female mice. High-resolution nano-computed tomography (nano-CT) analysis of frontal bone (**A**–**E**), parietal bone (**F**–**J**), and mandible (**K**–**O**) in 2-month-old female control (CTR, n = 5), heterozygous (Chet, n = 4), and conditional knockout (CKO, n = 5) mice. Representative cross-sectional images (**A**, **F**, **K**) show increased bone thickness in CKO mice. Quantitative analysis revealed significant increases in thickness (**B**, **G**, **L**) and bone volume (**C**, **H**, **M**) in all three bones of CKO mice compared to controls. Bone volume fraction (BV/TV) was significantly higher in frontal (**D**) and parietal bones (**I**) but decreased in the mandible (N), consistent with increased porosity. Tissue mineral density (TMD) was unchanged in the frontal bone (**E**), showed only minimal alteration in the parietal bone (**J**), and was significantly reduced in the mandible (**O**) of CKO mice. Data are mean ± SD. One-way ANOVA with Tukey’s post hoc test; *p < 0.05, **p < 0.01, ***p < 0.001, ****p < 0.0001, ^#^0.05 < *p* < 0.1. Corresponding data for male mice are shown in Fig. S1

Nano-computed tomography was used to quantitatively assess craniofacial bones, including the frontal bone, parietal bone, and mandible. No significant differences were observed between control and Chet mice across any parameters or bones. Compared to controls, conditional knockout (CKO) mice exhibited significantly increased bone acquisition in all three regions in both female (Fig. 2) and male (Fig. S1) mice. However, the magnitude and characteristics of these changes varied substantially across different bones.

In the frontal and parietal bones, CKO mice showed consistent greater bone thickness (Figs. 2A, B, G and S1A, E) and bone volume (Figs. 2C, H and S1B, F), accompanied by higher bone volume fraction (Figs. 2D, I and S1C, G), without changes or with only minimal changes in tissue mineral density (Figs. 2E, J and S1D, H). In contrast, the mandible displayed a distinct pattern: while bone thickness (Figs. 2L and S1I) and bone volume (Figs. 2M and S1J) were elevated, bone volume fraction was significantly less (Figs. 2N and S1K), likely due to greater porosity (Fig. 2K). Additionally, mandibular bone in CKO mice showed a significant reduction in tissue mineral density (Figs. 2O and S1L), unlike the other craniofacial bones.

**Fig. 3:**
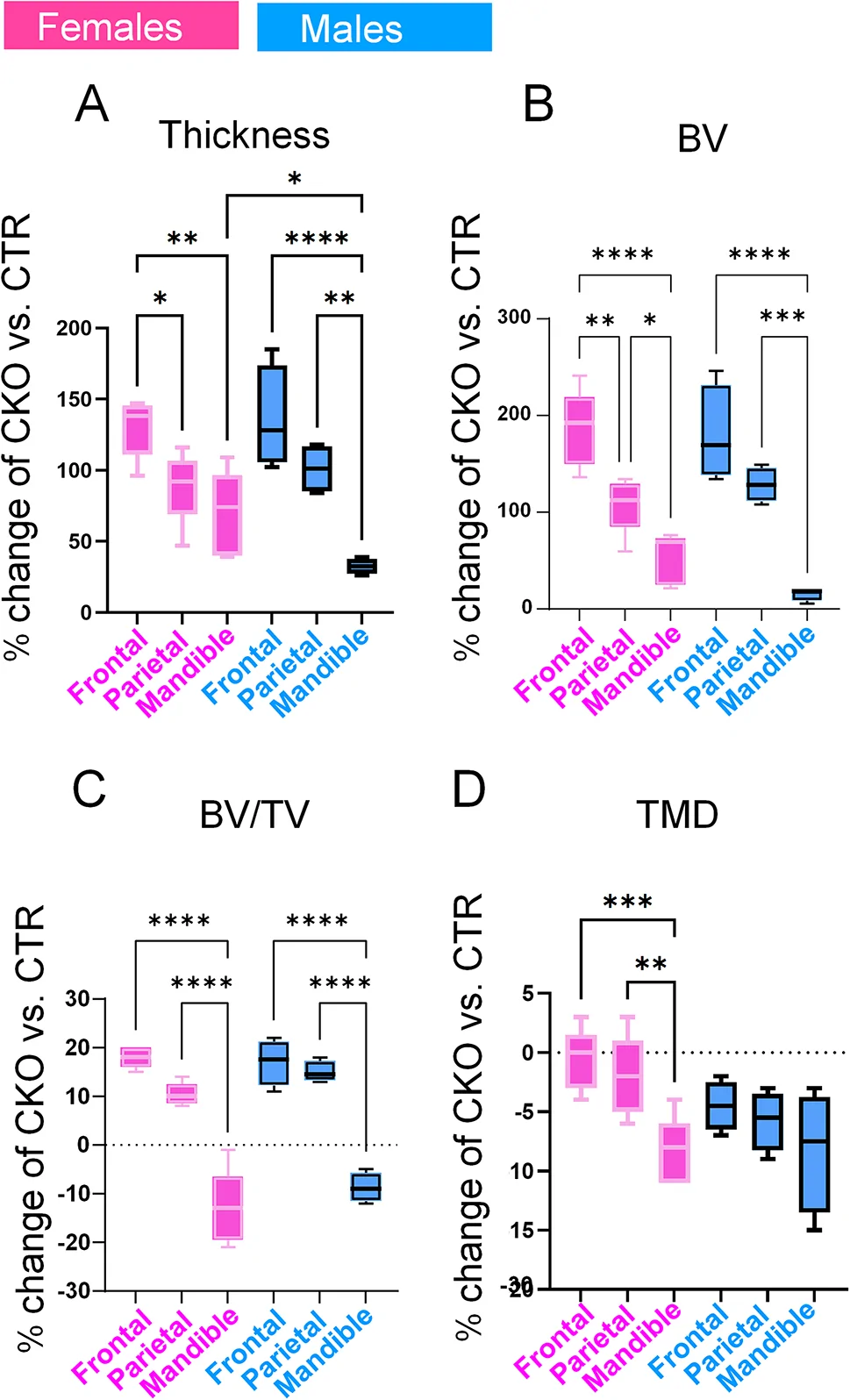
Comparison of *Tsc1* deletion-induced changes among craniofacial bones. Percent change in **A** thickness, **B** bone volume (BV), **C** bone volume fraction (BV/TV), and **D** tissue mineral density (TMD) in CKO mice relative to CTR for frontal bone, parietal bone, and mandible in females (pink) and males (blue). Percent change was calculated as (CKO − CTR)/CTR × 100%. Two-way ANOVA with Tukey’s post hoc test. *p < 0.05, **p < 0.01, ***p < 0.001, ****p < 0.0001. This differential response is unlikely due to location-specific Cre activity, as similar percentages of tdTomato-positive osteocytes and osteoblasts were observed across the three regions in Dmp1-Cre;Ai14 reporter mice (Fig. S2A, B). However, osteocyte density was significantly higher in the frontal bone compared to the parietal bone and mandible (Fig. S2C), which may partially explain the greater anabolic response in this region

To better visualize the bone-specific effects of *Tsc1* deletion, we calculated the percentage changes in each parameter for CKO mice relative to controls (Fig. 3). This analysis revealed that the frontal bone exhibited the largest relative elevation in both thickness and bone volume, while the mandible showed the smallest elevation (Fig. 3A, B). The parietal bone displayed intermediate changes. For bone volume fraction, the direction and magnitude of change also differed by region: both the frontal and parietal bones showed modest elevations, whereas the mandible showed a significant reduction (Fig. 3C). A similar bone-specific divergence was observed in tissue mineral density, which remained unchanged in frontal and parietal bones but was significantly reduced in the mandible, particularly in female mice (Fig. 3D).

These findings underscore the anatomical location-specific heterogeneity in the skeletal response to *Tsc1* deletion. This differential response is unlikely to be due to location-specific Cre activity, as similar percentages of tdTomato-positive osteocytes and osteoblasts were observed across the three regions in Dmp1-Cre;Ai14 reporter mice (Fig. S2A, B). However, osteocyte density was significantly higher in the frontal bone compared to the parietal bone and mandible (Fig. S2C). Altogether, these data suggest that local anatomical or cellular contexts—such as osteocyte density—may modulate the effects of mTORC1 signaling on bone acquisition.

### Deletion of *Tsc1* by 8-kb Dmp1-Cre Leads to Greater Trabecular and Cortical Bone Mass in the Femur

**Fig. 4:**
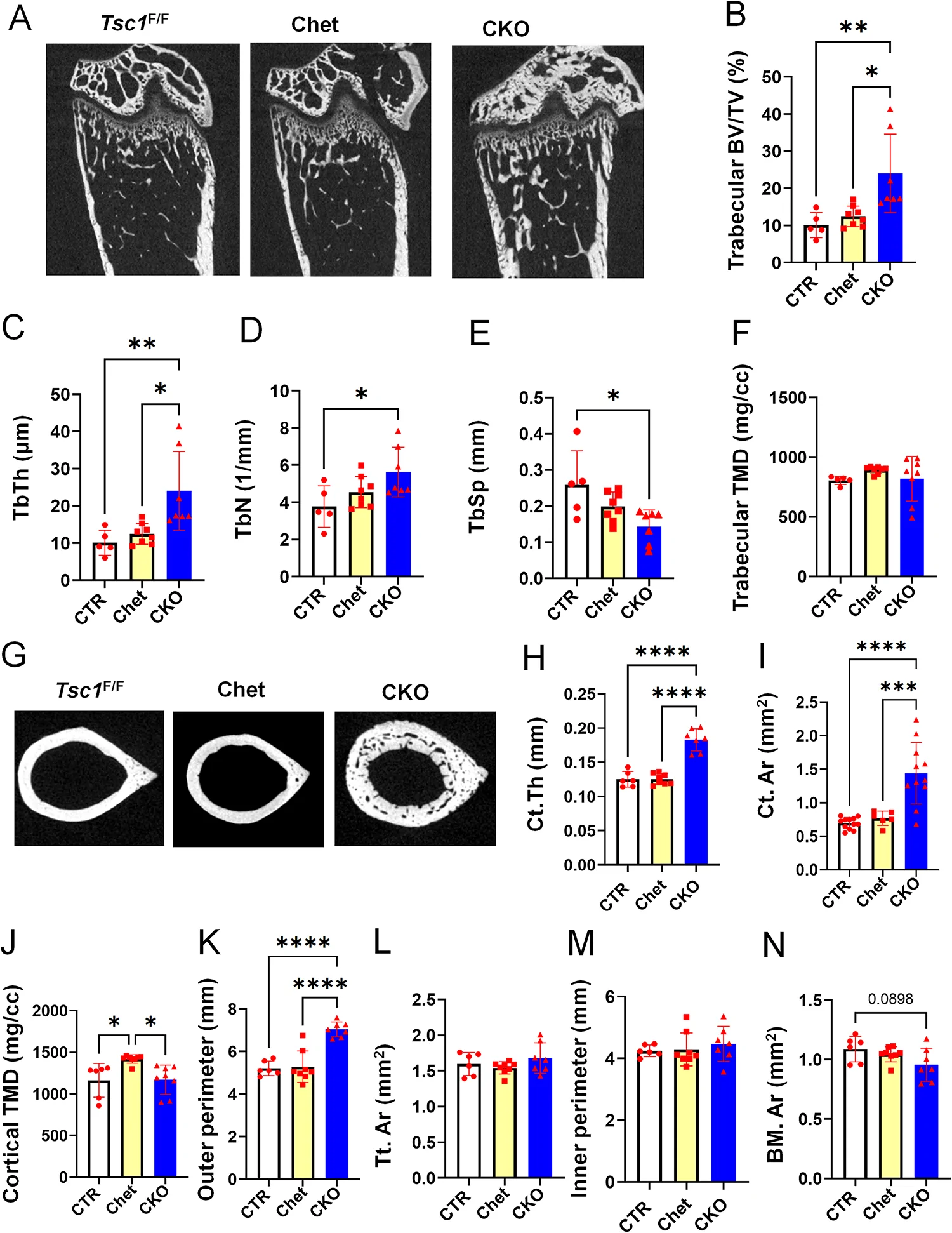
*Tsc1* deletion by 8-kb Dmp1-Cre augments femoral bone mass in female mice. Nano-CT analysis of distal femur in CTR (n = 6), Chet (n = 8), and CKO (n = 7) mice. **A** Representative longitudinal nano-CT images of distal femoral trabecular bone. Trabecular parameters include **B** bone volume fraction (BV/TV), **C** trabecular thickness (Tb.Th), **D** trabecular number (Tb.N), **E** trabecular separation (Tb.Sp), and **F** trabecular tissue mineral density (TMD). **G** Representative cross-sectional nano-CT images of the femoral mid-diaphysis. Cortical parameters include **H** cortical thickness (Ct.Th), **I** cortical area (Ct.Ar), **J** cortical TMD, **K** outer perimeter, **L** total cross-sectional area (Tt.Ar), **M** inner perimeter, and **N** marrow area (Ma.Ar). Data are presented as mean ± SD. *p < 0.05, **p < 0.01, ***p < 0.001, ****p < 0.0001; exact *p* values are indicated where applicable. Similar trends were observed in male mice (Fig. S3)

To investigate the effects of *Tsc1* deletion in non-craniofacial bones, we first analyzed the femur in both female (Fig. 4) and male (Fig. S3) mice. In the trabecular compartment, CKO mice exhibited a greater bone mass (Figs. 4A and S3), shown by the increase in bone volume fraction (Figs. 4B and S3A), trabecular thickness (Figs. 4C and S3B), and trabecular number (Figs. 4D and S3C), accompanied by a significant reduction in trabecular spacing (Figs. 4E and S3D). These changes occurred without alterations in trabecular tissue mineral density (Figs. 4F and S3E).

In the cortical compartment, CKO mice showed significantly augmented bone mass (Figs. 4G, S3 and S4) shown by the greater cortical thickness (Figs. 4H and S3F) and cortical area (Figs. 4I and S3G), with no significant changes in cortical tissue mineral density (Figs. 4J and S3H). In female CKO femurs, the outer perimeter was significantly larger (Fig. 4K), whereas total cross-sectional area remained unchanged (Fig. 4L). This discrepancy was explained by the rougher surface of CKO bone (Fig. 4G). Noticably, there are increased porosity in the cortical bone of CKO mice shown by the nanoCT image (Fig. 4G). Histological analysis showed that the increased porosity in thickened femoral cortical bone in CKO mice is characterized by expanded vascular-like spaces within the cortical matrix (Fig. S4).

**Fig. 5:**
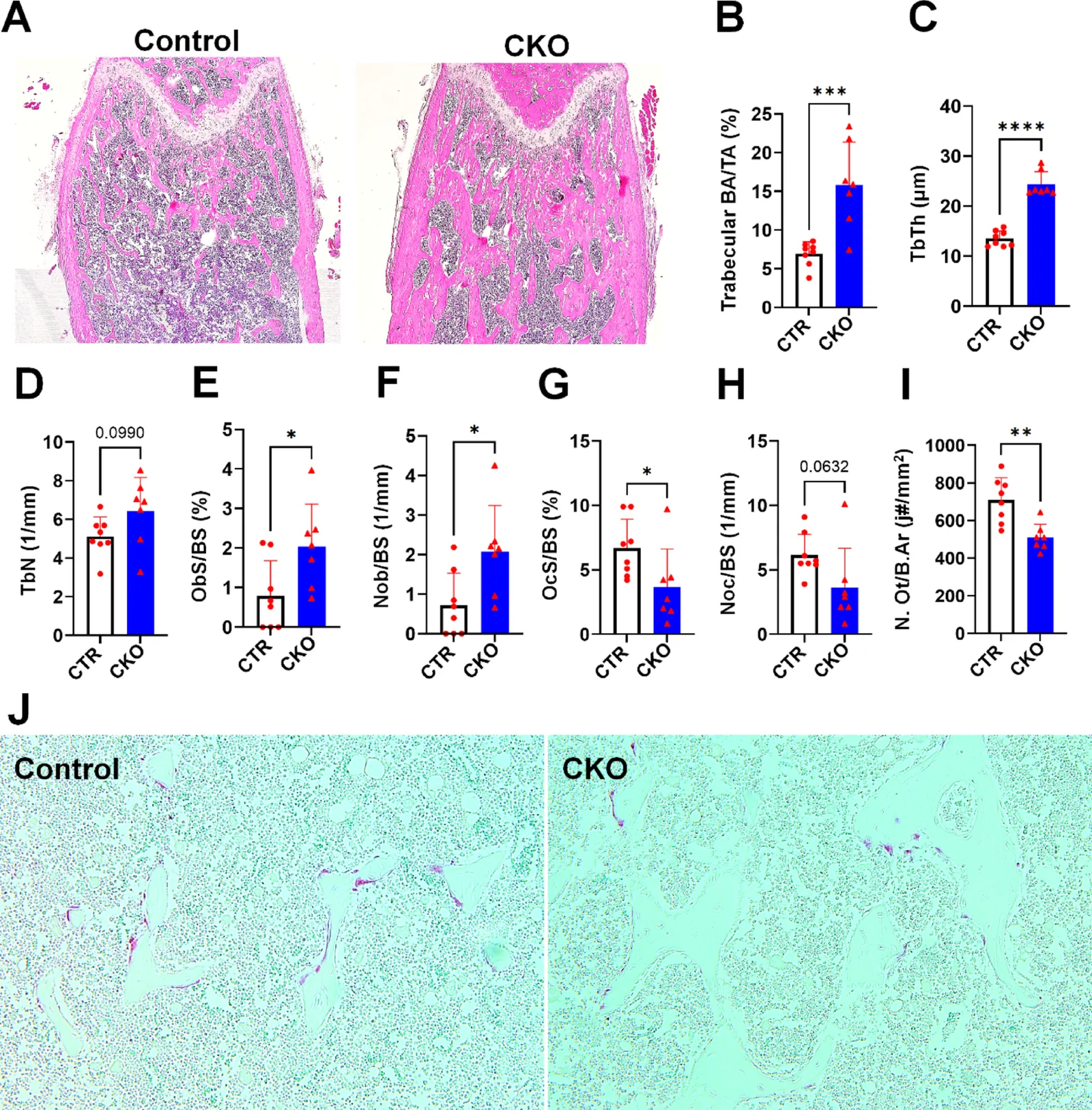
Histomorphometric analysis of femoral trabecular bone following *Tsc1* deletion by 8-kb Dmp1-Cre. **A** Representative histological sections of distal femoral trabecular bone from 2-month-old female CTR and CKO mice. **B**–**I** Histomorphometric quantification of trabecular bone parameters and bone cell indices, including trabecular bone area (**B**), trabecular thickness (**C**), trabecular number (**D**), osteoblast surface (**E**), osteoblast number (**F**), osteoclast surface (**G**), osteoclast number (**H**), and osteocyte number (**I**). **J** Representative TRAP staining showing reduced osteoclast presence in CKO mice. Data are presented as mean ± SD. *p < 0.05, **p < 0.01, ***p < 0.001, ****p < 0.0001; exact p values indicated where applicable

To determine the cellular mechanism of the augmented bone mass in CKO mice, we performed histological analysis on the femoral trabecular bone (Fig. 5). Consistent with the nanoCT analysis, there is greater trabecular bone volume in CKO mice (Fig. 5A, B), which is mainly contributed by greater trabecular thickness (Fig. 5C) and to lesser extent by greater trabecular number (Fig. 5D). CKO bone had greater osteoblast surface (Fig. 5E) and osteoblast number (Fig. 5F) with less osteoclast surface (Fig. 5G, J), osteoclast number (Fig. 5H, J) and osteocyte number (Fig. 5I), suggesting that the greater femoral trabecular bone mass in CKO mice could be contributed by both enhanced bone formation and less bone resorption.

We previously showed the high bone mass phenotype in mice lacking *Tsc1* in osterix-expressing cells [8]. To understand the potential difference between the deletion in osteroprogenitor cells by Osterix-Cre and the deletion in mature osteoblasts and osteocytes, we compared the the extent of bone parameter changes between two models. Our analysis of the femur nanoCT data showed that both deletion models had the similar extent of effect on trabecular bone parameters including bone volume fraction (Fig. S5A), trabecular thickness (Fig. S5B), trabecular number (Fig. S5C), trabecular spacing (Fig. S5D), and trabecular TMD (Fig. S5E). However, there are differences in the effects on femoral cortical bones between two deletion models. Deletion by Dmp1-Cre led to less elevation in cortical thickness (Fig. S6A), cortical area (Fig. S6B), and total area (Fig. S6C) while it led to higher increase in outer perimeter (Fig. S6D) and no difference in inner perimeter (Fig. S6E), marrow area (Fig. S6F), and cortical TMD (Fig. S6G).

### *Tsc1* Deletion by 8-kb Dmp1-Cre Increases Vertebral Bone Acquisition with Compartment-Specific Effects

**Fig. 6:**
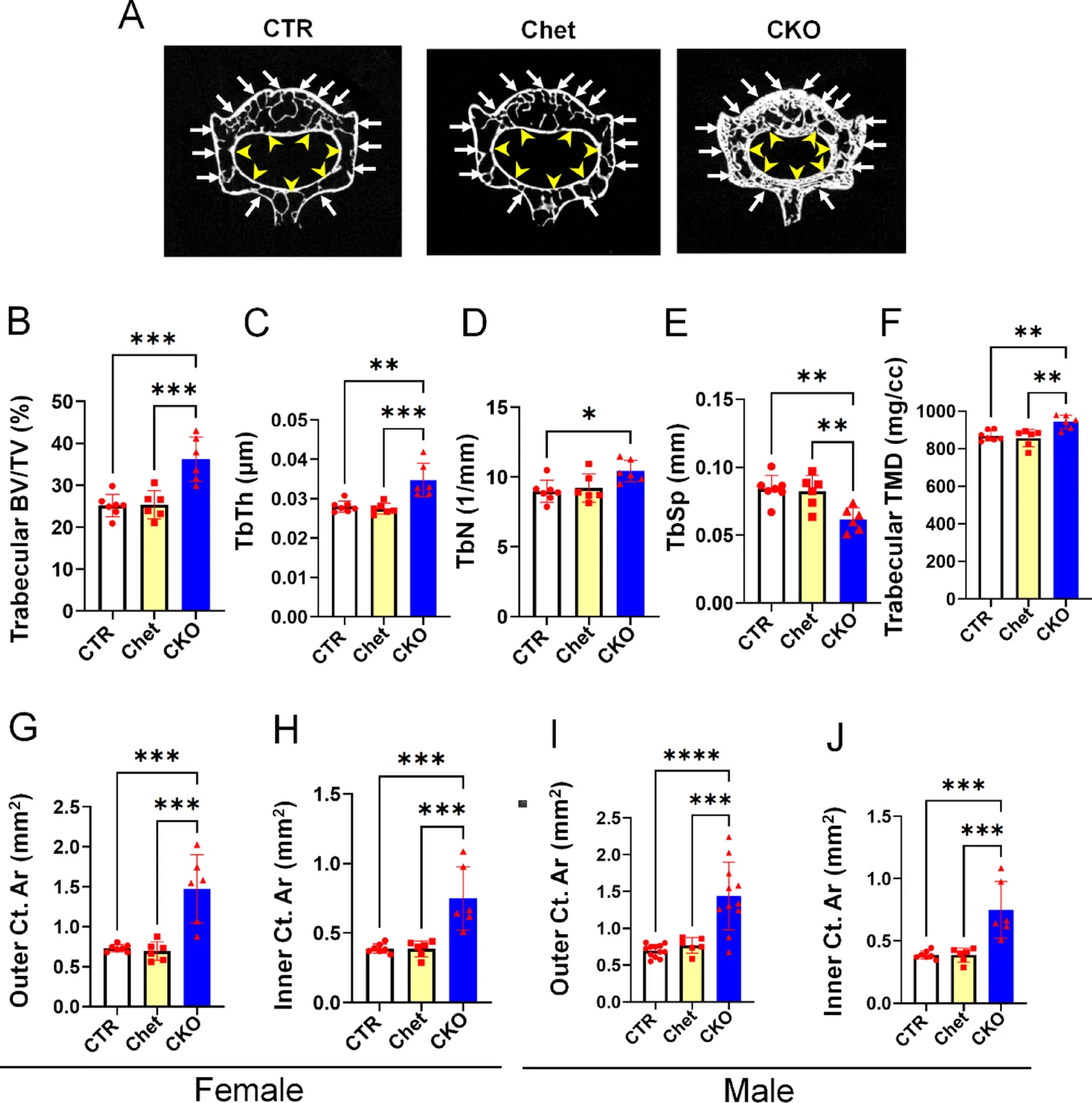
*Tsc1* deletion by 8-kb Dmp1-Cre augments vertebral bone mass in both trabecular and cortical compartments. Analysis of the third lumbar vertebra in CTR (n = 7), CHet (n = 6), and CKO (n = 6) mice. Trabecular parameters: **A** BV/TV, **B** Tb.Th, **C** Tb.N, **D** Tb.Sp, **E** trabecular TMD. CKO mice showed significant increases in BV/TV, Tb.Th, Tb.N, decreased Tb.Sp, and elevated TMD. Cortical parameters: **F** and **H** outer cortical area (Outer Ct.Ar) and **G** and **I** inner cortical area (Inner Ct.Ar), both markedly increased (> 100% in several comparisons) in CKO mice of both sexes. Data are mean ± SD. *p < 0.05, **p < 0.01, ***p < 0.001, ****p < 0.0001

We next analyzed the third lumbar vertebra to assess whether *Tsc1* deletion by 8-kb Dmp1-Cre has similar effects in axial skeletal sites. Our data showed a significant higher vertebral bone mass in CKO mice (Fig. 6A), the elevation in trabecular bone volume fraction (Figs. 6B and S7A) is associated with higher trabecular thickness (Figs. 6C and S7B), higher trabecular number (Figs. 6D and S7C), and lower trabecular spacing (Figs. 6E and S7D). Notably, this trabecular bone mass elevation was accompanied by a greater tissue mineral density (Figs. 6F and S7E). Importantly, the impact of *Tsc1* deletion on the cortical compartment of the vertebra was even more pronounced than in the trabecular compartment. Both outer cortical area and inner cortical area elevated by more than 100% in CKO mice of both sexes (Fig. 6G–J), indicating a compartment-specific enhancement of bone acquisition in the axial skeleton.

**Fig. 7:**
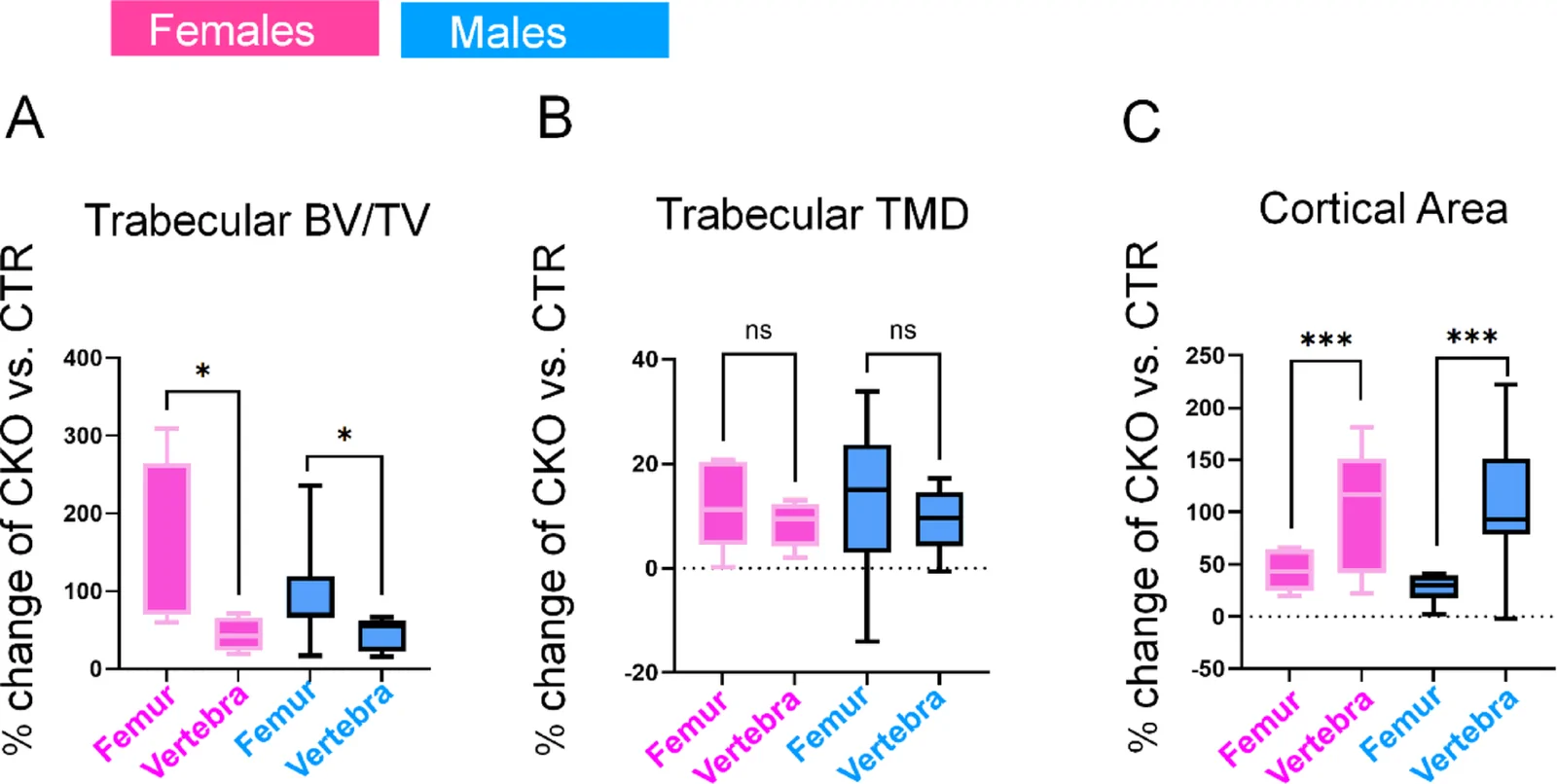
Comparison of *Tsc1* deletion-induced changes between femur and vertebra. Percent changes in CKO vs. CTR mice for **A** BV/TV, **B** trabecular TMD, and **C** cortical area (Ct.Ar) in femur and vertebra for both sexes. BV/TV increase was significantly greater in the femur, TMD changes were comparable between sites, and Ct.Ar increase was greater in the vertebra. *p < 0.05; ***p < 0.001; ns, not significant

To further compare the skeletal site-specific effects of *Tsc1* deletion, we quantified the percent change in trabecular bone volume fraction, tissue mineral density, and cortical bone area in the femur and vertebra of CKO mice relative to controls (Fig. 7). Although there is no difference between male and female mice in both trabecular and cortical parameters in femur and vertebrae (Fig. S8), The elevation in trabecular bone volume fraction was significantly greater in the femur than in the vertebra in both sexes (Fig. 7A), while the percent elevation in trabecular tissue mineral density was comparable between sites (Fig. 7B). In contrast, the elevation in cortical bone area was significantly greater in the vertebra than in the femur in both sexes (Fig. 7C).

Together, these findings demonstrate a bone-specific and compartment-specific effect of *Tsc1* deletion by Dmp1-Cre. While femoral bone exhibited a stronger response in the trabecular compartment, vertebral bone showed a more robust elevation in the cortical compartment, highlighting spatial and structural heterogeneity in skeletal responses to *Tsc1* deletion.

### *Tsc1* Deletion by 8-kb Dmp1-Cre Enhances mTORC1 Activity

**Fig. 8:**
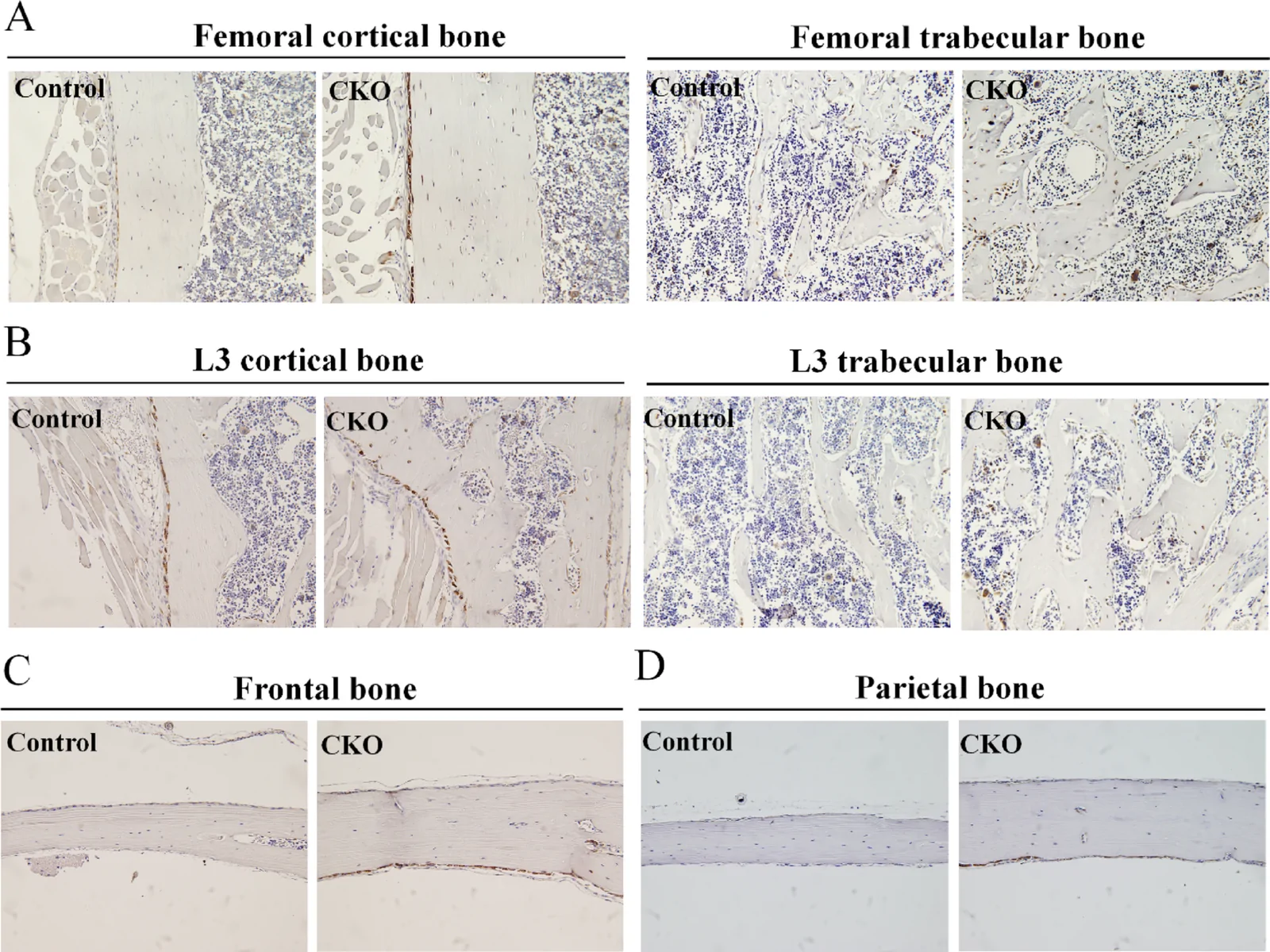
Enhanced mTORC1 signaling in multiple skeletal sites following *Tsc1* deletion. Representative immunohistochemical staining for phosphorylated S6 (p-S6), a downstream readout of mTORC1 activity, in 2-month-old female control and CKO mice. **A** Femur cortical and trabecular bone. In cortical bone, p-S6 staining is increased in both osteoblasts and osteocytes in CKO mice. In trabecular bone, CKO samples show enhanced p-S6 immunoreactivity compared to controls. **B** L3 vertebra cortical and trabecular bone. In cortical bone, osteoblast staining is prominent in both groups, while osteocyte staining is more evident in CKO mice. In vertebral trabecular bone, p-S6 staining is enhanced in CKO mice relative to controls. **C** and **D** Frontal and parietal bones. In both cranial sites, p-S6 immunoreactivity is primarily observed in osteoblasts, with stronger staining in CKO samples, whereas osteocyte staining remains low and comparable between genotypes

TSC1 is a key negative regulator of mTORC1 signaling. To determine whether *Tsc1* deletion by 8-kb Dmp1-Cre enhances mTORC1 activity, we performed immunostaining for phosphorylated S6 (p-S6), a downstream readout of mTORC1 activation. As shown in Fig. 8, p-S6 immunoreactivity was higher in CKO mice compared with controls across multiple skeletal sites and compartments. In the femur, stronger p-S6 staining was observed in both cortical and trabecular bone, with enhanced signals in osteoblasts and osteocytes in CKO mice (Fig. 8A). In the L3 vertebra, control samples exhibited prominent p-S6 staining in cortical osteoblasts, limiting detection of further changes in this population; however, osteocyte staining was more apparent in CKO mice (Fig. 8B, left panels). In vertebral trabecular bone, p-S6 immunoreactivity was also elevated in CKO mice relative to controls (Fig. 8B, right panels). In the frontal (Fig. 8C) and parietal (Fig. 8D) bones, p-S6 staining was primarily detected in osteoblasts and was more intense in CKO samples, whereas osteocyte staining remained low and comparable between genotypes. Together, these findings confirm robust activation of mTORC1 signaling in Dmp1-Cre–targeted cells following Tsc1 deletion across both appendicular and axial skeletal sites.

Given previous reports showing that mTORC1 activation can suppress sclerostin expression in osteocytes [9], we also examined sclerostin by immunostaining (Fig. S9). Consistent with published findings in femoral cortical bone, sclerostin staining appeared reduced in femoral osteocytes of CKO mice. In contrast, baseline sclerostin staining was relatively low in L3 vertebrae and calvarial bones under identical staining conditions, limiting our ability to determine whether *Tsc1* deletion alters sclerostin expression at these sites. Together, these data demonstrate robust mTORC1 activation across skeletal compartments and site-specific patterns of downstream response.

## Discussion

Dmp1-Cre is widely used to target osteocytes [10]. The original 10-kb Dmp1-Cre line drives recombination in osteocytes, osteoblasts, and multiple non-skeletal tissues, prompting the development of the 8-kb Dmp1-Cre with improved specificity [14]. Nonetheless, previous work [11] and our current results show that the 8-kb version still targets many osteoblasts on the bone surfaces. We also observed recombination in skeletal muscle but not in odontoblasts, whereas the 10-kb line has broader activity, including in odontoblasts and other visceral organs. Our previous work also demonstrated ectopic activity of the 8-kb Dmp1-Cre in non-skeletal tissues [22]. Despite this ectopic expression, the 8-kb Dmp1-Cre remains a valid tool to target mature osteoblast-lineage cells, including osteocytes, for skeletal studies.

Our data reveal pronounced anatomical location-specific effects of *Tsc1* deletion. In craniofacial bones, frontal and parietal bones exhibited parallel elevations in thickness, volume, and bone volume fraction, whereas the mandible showed higher volume but lower bone volume fraction and tissue mineral density. Reporter analysis confirmed similar recombination efficiency across sites, suggesting that intrinsic features—such as higher osteocyte density in frontal bone—and differences in developmental origin, loading environment, or remodeling activity likely contribute to these site-dependent responses. We also observed bone compartment-specific differences in response to *Tsc1* deletion. In the femur, *Tsc1* deletion augmented both trabecular and cortical bone mass, whereas in the vertebra, cortical bone area elevated more than 100%—a far greater response than in the trabecular compartment. Using phosphorylated S6 (p-S6) as a downstream readout of mTORC1 signaling, we observed elevated p-S6 immunoreactivity across multiple skeletal sites and compartments in CKO mice, indicating effective mTORC1 activation. The comparable mTORC1 activation suggests that there are unidentified local or lineage-dependent mechanisms contributing to the differential responses following *Tsc1* deletion at different skeletal sites and compartments.

Comparisons with our previous study using Osterix-Cre to delete *Tsc1* further highlight target cell stage-dependent and compartment-specific effects. In the initial work, which focused on femoral trabecular bone at 1 month of age, *Tsc1* deletion in osteoblast progenitors reduced trabecular bone mass [7], while our additional data from the same cohort revealed a concomitant elevation in cortical bone mass [8]. This early divergence between trabecular and cortical compartments indicates that compartment-specific responses to mTORC1 activation occur not only in mature osteoblast/osteocyte-targeted models but also when targeting osteoprogenitors. To further contextualize our findings, we compared the skeletal phenotype of 8-kb Dmp1-Cre and Osterix-Cre *Tsc1* deletion models at the same postnatal age (2 months), in the same sex (female), and within the same skeletal site (femur). While trabecular parameters changes compared to their respective controls are largely comparable between two models (Fig. S5), deletion of *Tsc1* using Osterix-Cre produced more pronounced cortical changes, including greater elevation in cortical thickness, cortical area and total cross-sectional area (Fig. S6). This difference suggests that loss of *Tsc1* in earlier osteoblast-lineage cells may exert broader effects on cortical bone than deletion restricted to more mature osteoblasts and osteocytes.

The lack of differences between control and heterozygous (CHet) mice in our study is consistent with our previous findings using the Osterix-Cre model [8], where *Tsc1* heterozygous deletion likewise did not alter bone mass in either calvarial or femoral cortical bone. These results suggest that a single functional *Tsc1* allele is generally sufficient to maintain skeletal homeostasis in osteoblast-lineage cells, at least under basal physiological conditions. Although complete loss of *Tsc1* produces marked mTORC1 hyperactivation and robust bone accrual, the heterozygous state appears to fall below the threshold required to elicit measurable skeletal changes in vivo.

Consistent with findings from the Osterix-Cre model [8], deletion of *Tsc1* by 8-kb Dmp1-Cre resulted in enhanced mTORC1 activity. In the Osterix-Cre model, elevated bone mass was not associated with compromised bone structure and was accompanied by improved mechanical properties. In the 8-kb Dmp1-Cre CKO mice, greater femoral cortical thickness was accompanied by the presence of small intracortical porous spaces. However, these features do not indicate hypomineralization or structural disorganization, as tissue mineral density was unchanged and histological analysis revealed otherwise normal bone architecture. The small intracortical spaces likely represent vascular structures, which may expand to accommodate the much greater cortical bone mass. However, the underlying mechanism remains to be determined.

Histomorphometric analysis indicates that the augmented femoral trabecular bone mass in CKO mice is attributable to both increased bone formation and reduced bone resorption. Our previous work showed that *Tsc1* deletion in neural crest-derived cells, including frontal bone osteoblasts, leads to greater frontal bone mass primarily through enhanced bone formation without changes in osteoclast parameters [4]. In contrast, deletion of *Tsc1* in Osterix-expressing cells results in reduced femoral trabecular bone mass at 1 month of age associated with lower osteoblast number and higher osteoclast number [7]. Notably, in that model, femoral trabecular bone mass was elevated at 2 months of age, and calvarial and femoral cortical bone mass is already elevated at 1 month of age [8]. Together, these findings suggest that the skeletal effects of *Tsc1* deletion are both site-specific and developmentally regulated.

Prior work using 10-kb Dmp1-Cre to delete *Tsc1* proposed that enhanced bone formation is associated with reduced sclerostin expression in osteocytes [9]. In our 8-kb Dmp1-Cre model, immunostaining in femoral cortical bone is consistent with a regulatory role of *Tsc1* in sclerostin expression. However, because baseline sclerostin staining was low in other skeletal sites, we cannot determine whether this mechanism operates uniformly across the skeleton. In the Osterix-Cre model, increased cortical proliferation has been reported [8] and may contribute to the more substantial cortical expansion observed relative to the 8-kb Dmp1-Cre model. Together, these comparisons highlight that the skeletal consequences of *Tsc1* deletion depend on both the developmental stage of the targeted lineage and the anatomical context of the bone.

In summary, *Tsc1* deletion in mature osteoblast-lineage cells using 8-kb Dmp1-Cre produces robust but heterogeneous bone mass elevation, shaped by skeletal region, compartment, and developmental stage of the targeted cells. These findings underscore the complexity of mTORC1 regulation in bone and highlight the need to consider anatomical location, compartmental context, and target cell stage when developing mTORC1-based anabolic strategies.

## Supplementary Information

Below is the link to the electronic supplementary material.

Supplementary Material 1
