# Supplementary material for: Region- and Compartment-Specific Elevation of Bone Mass in Mice Following Tsc1 Deletion in 8-kb Dmp1-Cre-Expressing Cells
Source: Calcif Tissue Int. 2026 Apr 27;117(1):66. doi: 10.1007/s00223-026-01532-8 (PMC13111507; doi:10.1007/s00223-026-01532-8)
Supplement: Supplementary file 1 — Supplementary Material 1 [file 223_2026_1532_MOESM1_ESM.pdf]

Region- and Compartment-Specific Elevation of Bone Mass In Mice Following *Tsc1* Deletion in 8-kb Dmp1-Cre–Expressing Cells

Iya Ghassib[1,2#], Anusha Inti[1#], Nushaba Hossain[1], Thomas Kim[1], Danielle Moon[1], Lu Han[1], Rawan Mohsen[1], Honghao Zhang[1], Nicholas Auyeung[1], Yuji Mishina[1], Daniela Mendonça[1,3], Darnell Kaigler[4], Teresita Bellido[5], Fei Liu[1*]

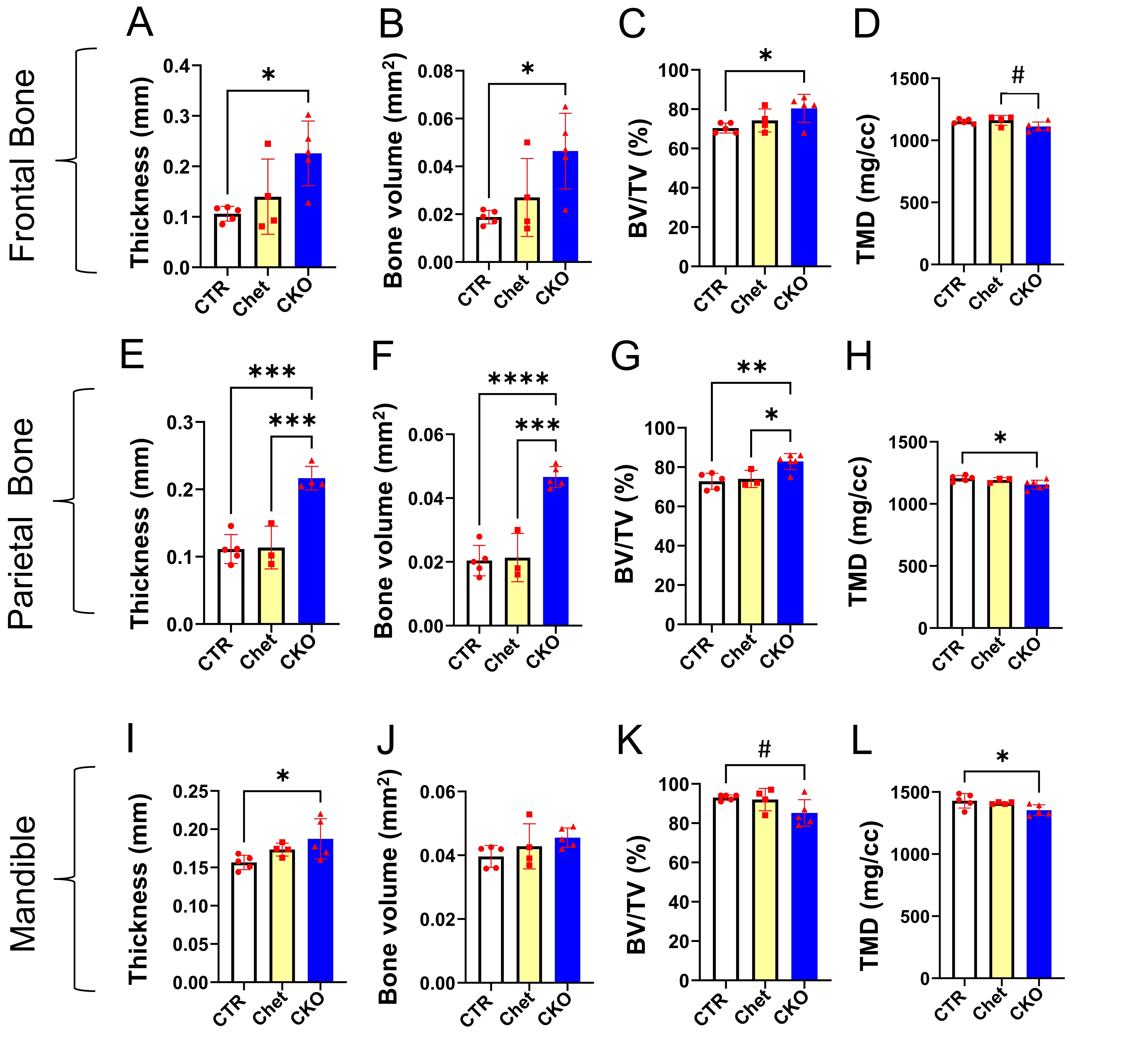

Figure S1. *Tsc1* deletion by Dmp1-Cre leads to greater craniofacial bone mass in male mice.

Nano-computed tomography analysis of the frontal bone (A–D), parietal bone (E–H), and mandible (I–L) in 2-month-old male mice with the indicated genotypes. Conditional knockout (CKO) mice ($n = 5$) exhibited significantly increased bone thickness (A, E, I) and bone volume (B, F, J) in all three craniofacial regions compared to control (CTR, $n = 5$; CHet, $n = 4$) groups. Bone volume fraction (BV/TV) was significantly increased in the frontal (C) and parietal (G) bones but reduced in the mandible (K). Tissue mineral density (TMD) showed minimal or no changes in the frontal (D), but was significantly decreased in the parietal bones (H) and mandible (L) of CKO mice. Data are mean ± SD. *$p < 0.05$, **$p < 0.01$, ***$p < 0.001$, ****$p < 0.0001$, #$p < 0.1$ but > 0.05 by one-way ANOVA with Tukey's post hoc test.

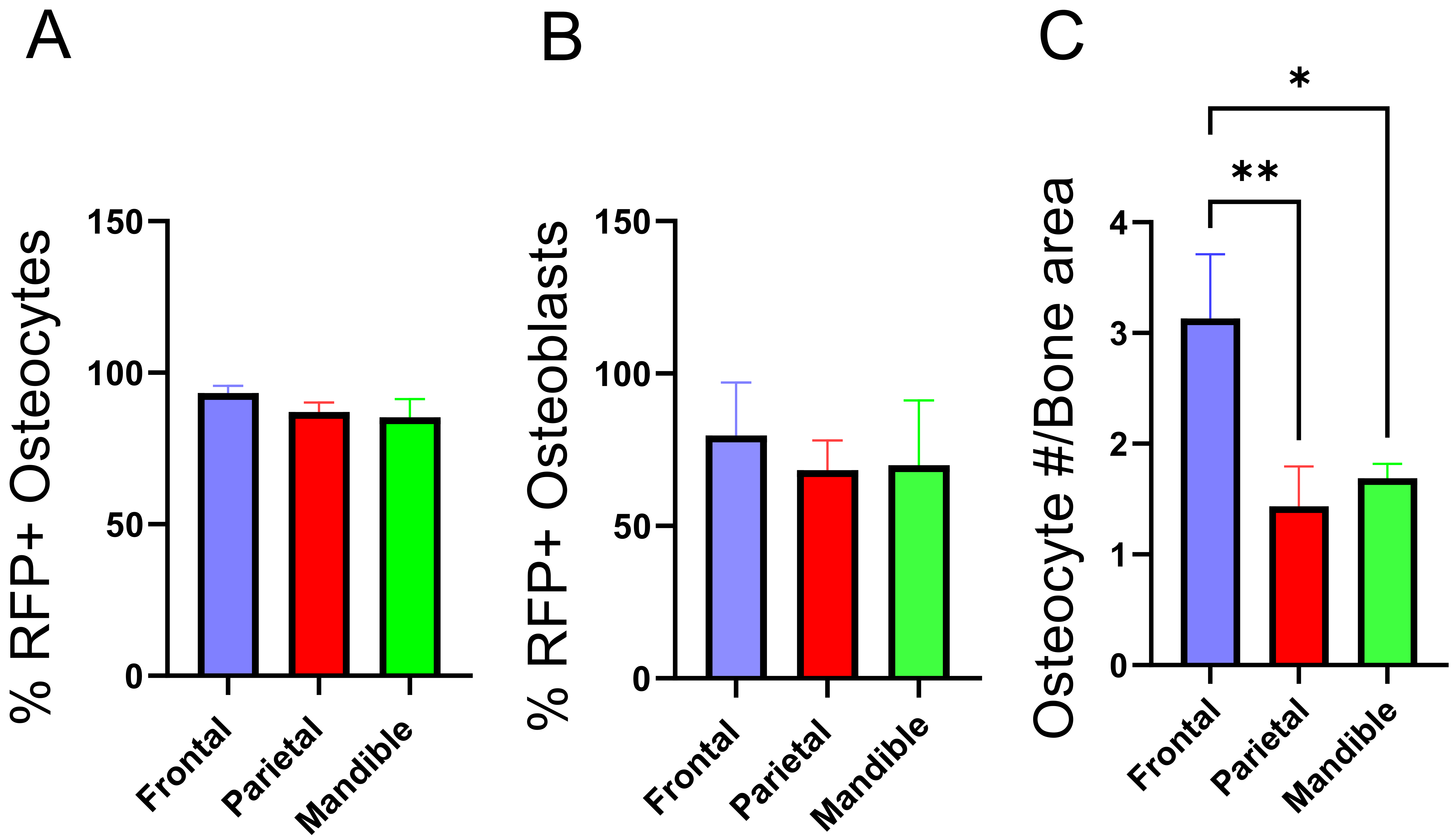

Figure S2. Comparison of DMP1-Cre activity in osteoblasts and osteocytes in different craniofacial bones.

Quantification of tdTomato-positive (RFP^+) cells in Dmp1-Cre;Ai14 reporter mice at 1 month of age, showing the percentage of osteocytes (A) and osteoblasts (B) labeled in the frontal bone, parietal bone, and mandible. RFP^+ cells were similarly abundant across regions for both osteocytes and osteoblasts, indicating that the differential effects of Tsc1 deletion on bone parameters (Fig. 3) are not attributable to region-specific Cre activity. (C) Osteocyte density (osteocyte number per bone area) was significantly higher in the frontal bone than in the parietal bone and mandible, which may contribute to the greater anabolic response observed in this region following *Tsc1* deletion. $^*p < 0.05$, $^{**}p < 0.01$; one-way ANOVA with Tukey’s post hoc test.

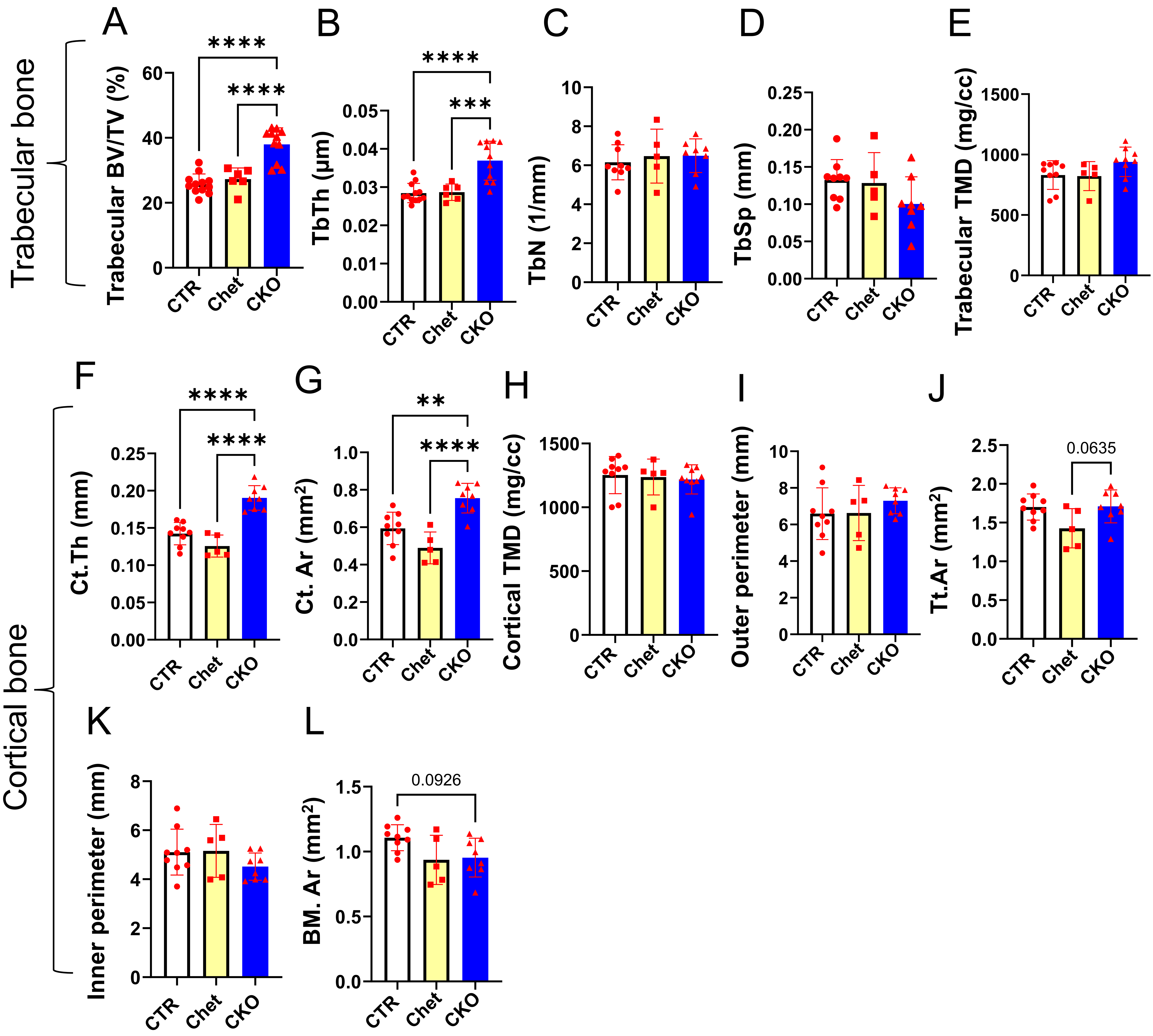

Figure S3. ***Tsc1* deletion by Dmp1-Cre leads to greater femoral bone mass in male mice.** Trabecular bone parameters in the distal femur of control (CTR), conditional heterozygous (Chet), and conditional knockout (CKO) mice: (A) bone volume fraction (BV/TV), (B) trabecular thickness (Tb.Th), (C) trabecular number (Tb.N), (D) trabecular separation (Tb.Sp), and (E) trabecular tissue mineral density (TMD). Cortical bone parameters in the mid-diaphysis: (F) cortical thickness (Ct.Th), (G) cortical area (Ct.Ar), and (H) cortical TMD. Periosteal geometry: (I) outer perimeter, and (J) total cross-sectional area (Tt.Ar). Endosteal geometry: (K) inner perimeter and (L) marrow area (Ma.Ar). CTR: n = 6; CHet: n = 8; CKO: n = 7. Data are mean ± SD. *$p < 0.05$, **$p < 0.01$, ***$p < 0.001$, ****$p < 0.0001$. Overall, male mice exhibited similar trends to females (Figure 4), indicating a consistent skeletal response to *Tsc1* deletion across sexes.

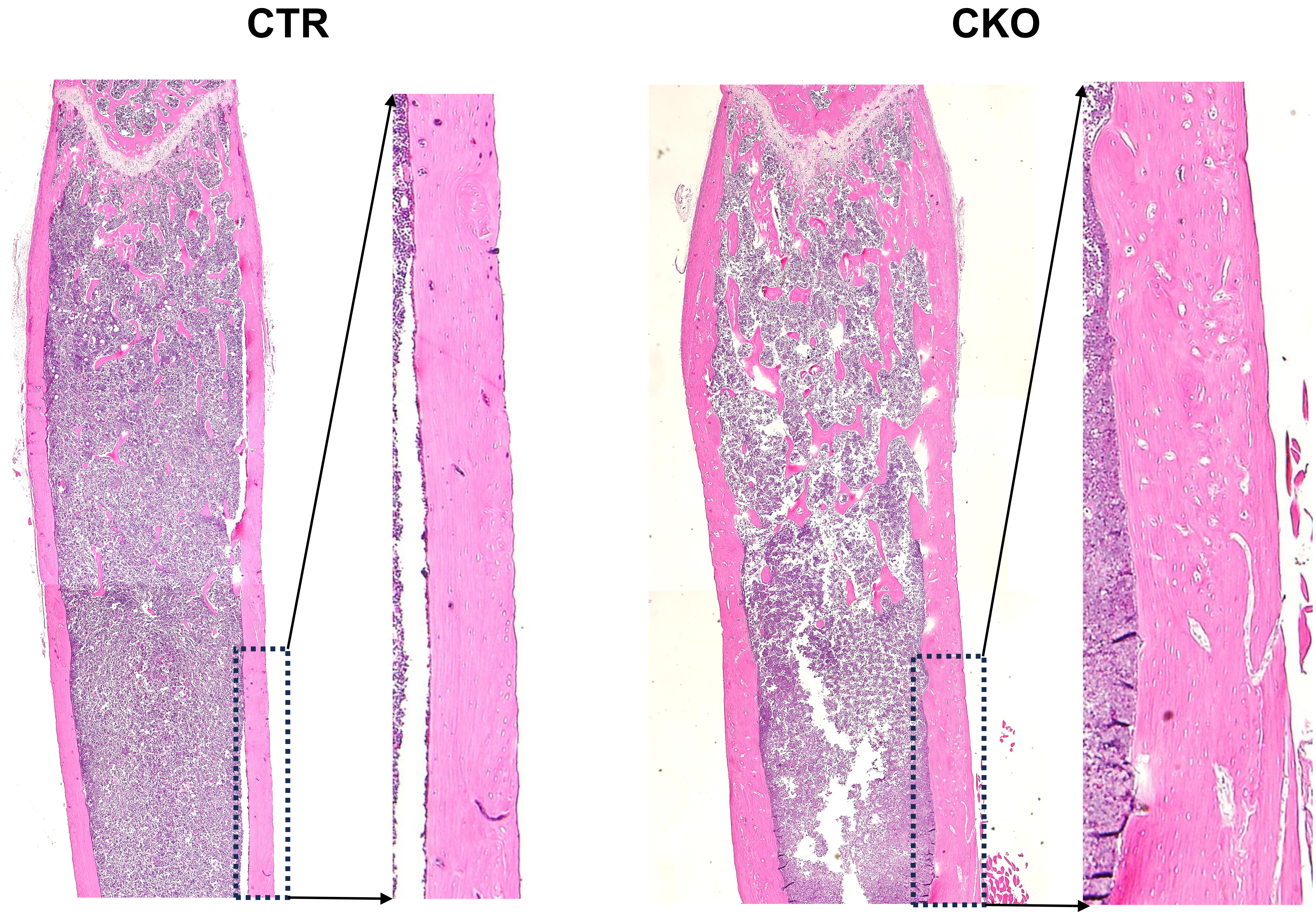

Fig. S4. Histological analysis of femoral cortical bone in 2-month-old male mice.

Representative hematoxylin and eosin–stained longitudinal sections of femurs from control (*Tsc1*$^{\text{flox/flox}}$) and conditional knockout (*Tsc1*$^{\text{flox/flox}}$; Dmp1-Cre) mice. Low-magnification images (left panels) show overall femoral morphology. Boxed regions indicate the mid-diaphyseal cortical bone area shown at higher magnification (right panels). Compared with controls, CKO mice exhibit visibly thickened cortical bone accompanied by increased intracortical porosity, characterized by expanded vascular- or canal-like spaces within the cortical matrix. These structural changes are consistent with the nano-CT findings of increased cortical bone mass and porosity in CKO mice.

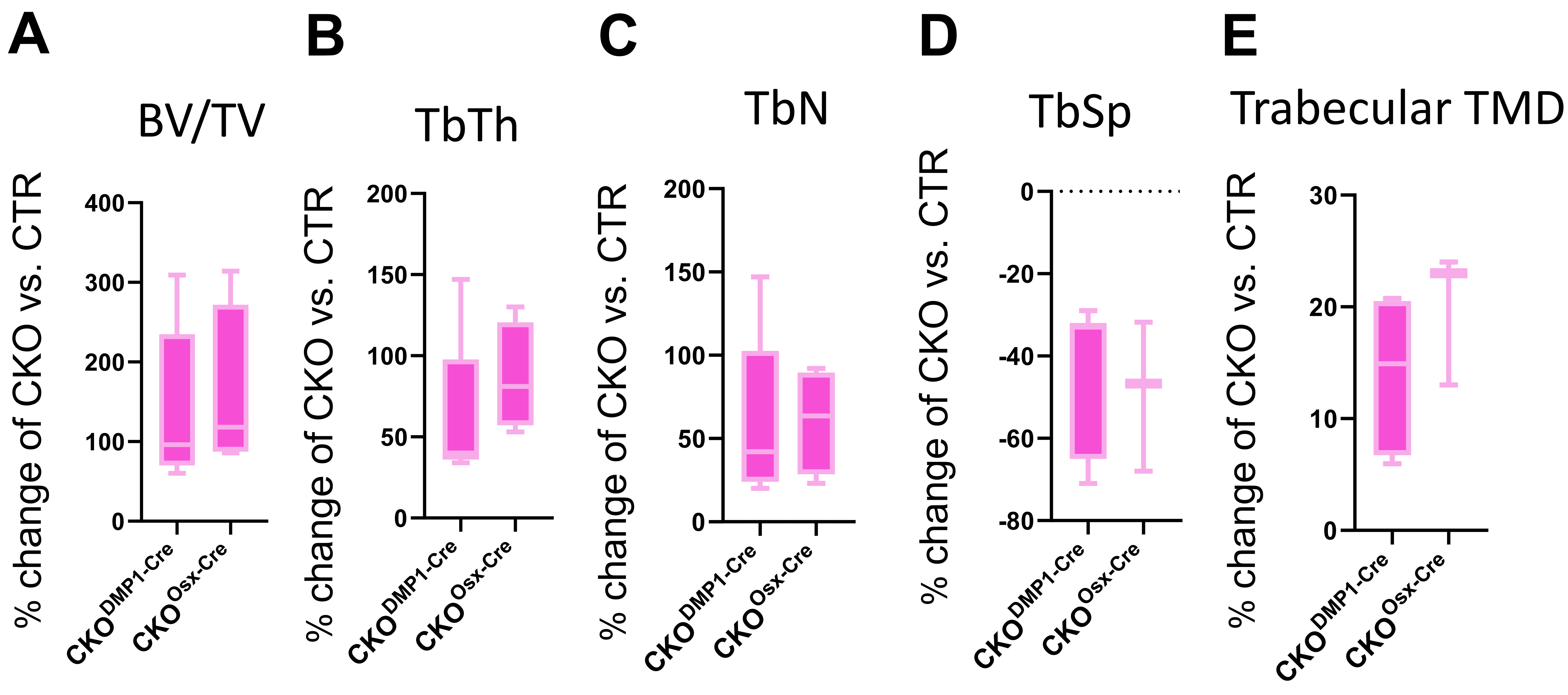

Fig. S5. Comparable *Tsc1* deletion–induced trabecular bone changes in femurs between Dmp1-Cre and Osx-Cre models. Percent changes in femoral trabecular bone parameters in female conditional knockout (CKO) mice relative to respective controls (CTR) at 2 months of age. Boxplots show CKO-vs-CTR percent changes for (A) trabecular bone volume fraction (BV/TV), (B) trabecular thickness (Tb.Th), (C) trabecular number (Tb.N), and (D) trabecular spacing (Tb.Sp), (E) tissue mineral density (TMD) in the 8-kb Dmp1-Cre model (left) and the Osx-Cre model (right). Both Cre drivers yielded comparable trabecular responses to *Tsc1* deletion. Data are presented as box-and-whisker plots. The calculation of percentage changes in Osx-Cre model are based on our previous publication (Bone. 2026 Feb:203:117695).

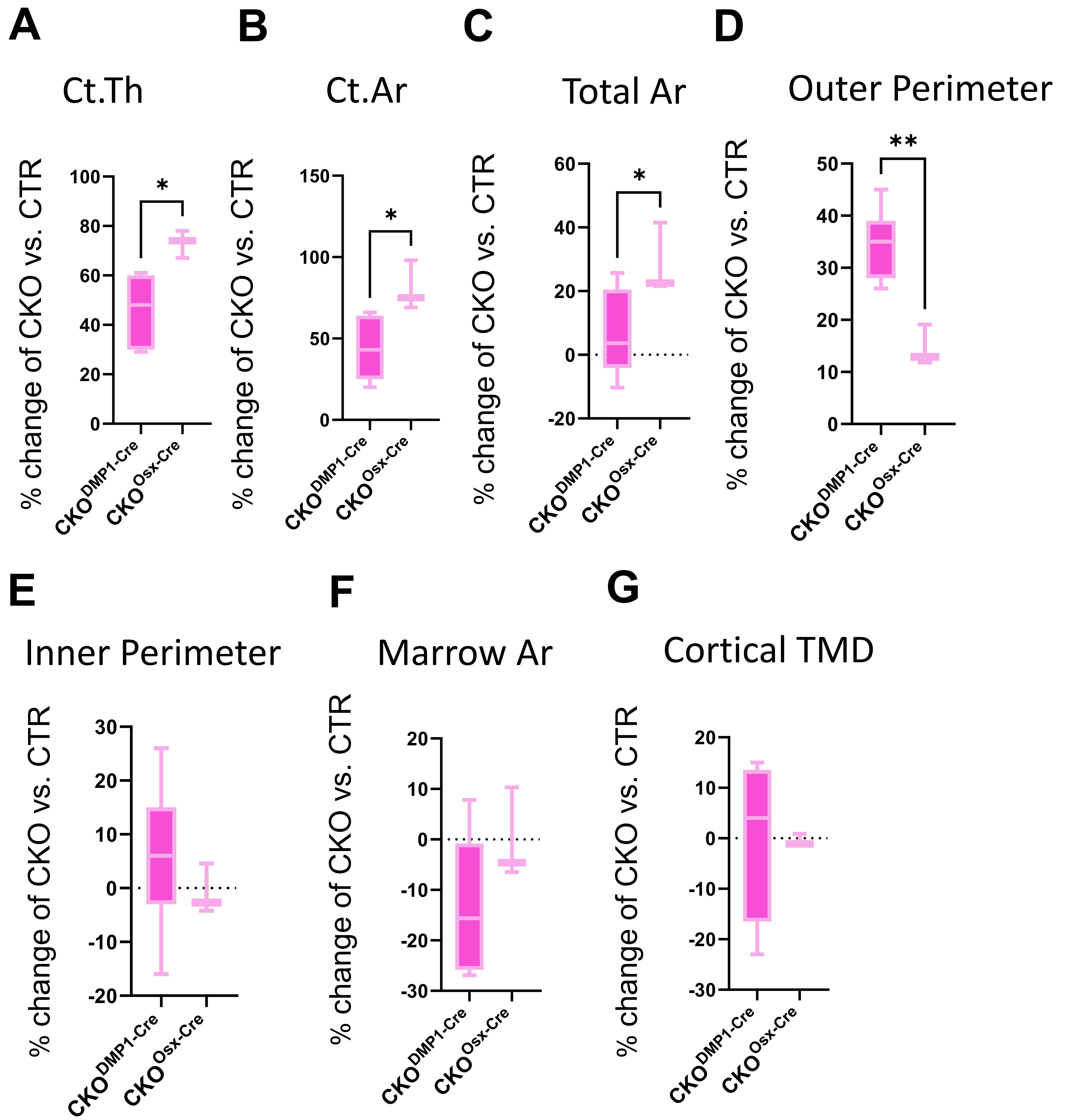

Fig. S6. Differential Tsc1 deletion–induced changes in femoral cortical bone of female mice between Dmp1-Cre and Osx-Cre models. Percent change of femoral cortical bone parameters in **female** conditional knockout (CKO) mice relative to respective female controls (CTR) at 2 months of age. Box-and-whisker plots show CKO-vs-CTR percent changes for (A) cortical thickness (Ct.Th), (B) cortical area (Ct.Ar), (C) total cortical area (Tt.Ar), (D) outer perimeter, (E) inner perimeter, (F) marrow area (Ma.Ar), and (G) cortical TMD in the 8-kb Dmp1-Cre model (left) and the Osx-Cre model (right). *$p < 0.05$. **$p < 0.01$. The calculation of percentage changes in Osx-Cre model are based on our previous publication (Bone. 2026 Feb:203:117695).

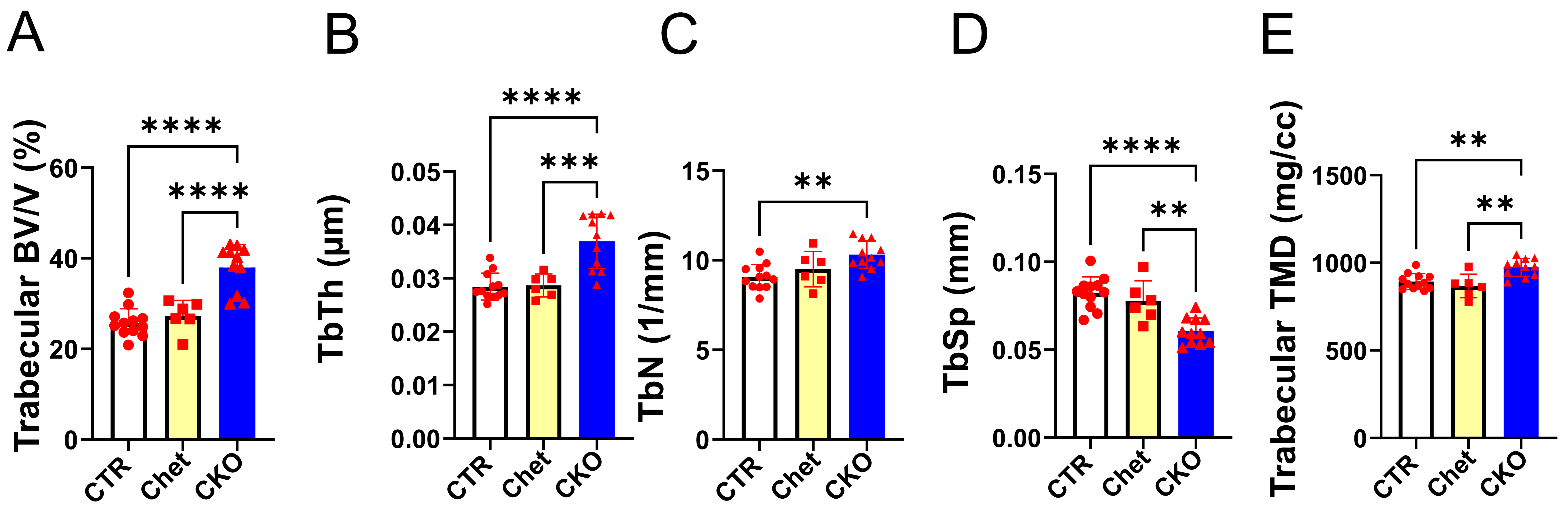

Figure S7. *Tsc1* deletion by Dmp1-Cre leads to greater vertebral trabecular bone mass in male mice.

(A–E) Trabecular bone parameters of the third lumbar vertebra in male control (CTR, n = 12), heterozygous (Chet, n = 6), and conditional knockout (CKO, n = 11) mice. CKO mice exhibited significantly higher bone volume fraction (BV/TV; A), trabecular thickness (Tb.Th; B), and trabecular number (Tb.N; C), with decreased trabecular spacing (Tb.Sp; D). Tissue mineral density (TMD) was also significantly elevated in CKO mice (E). Data are shown as mean ± SD. $*p < 0.05$, $**p < 0.01$, $***p < 0.001$, $****p < 0.0001$.

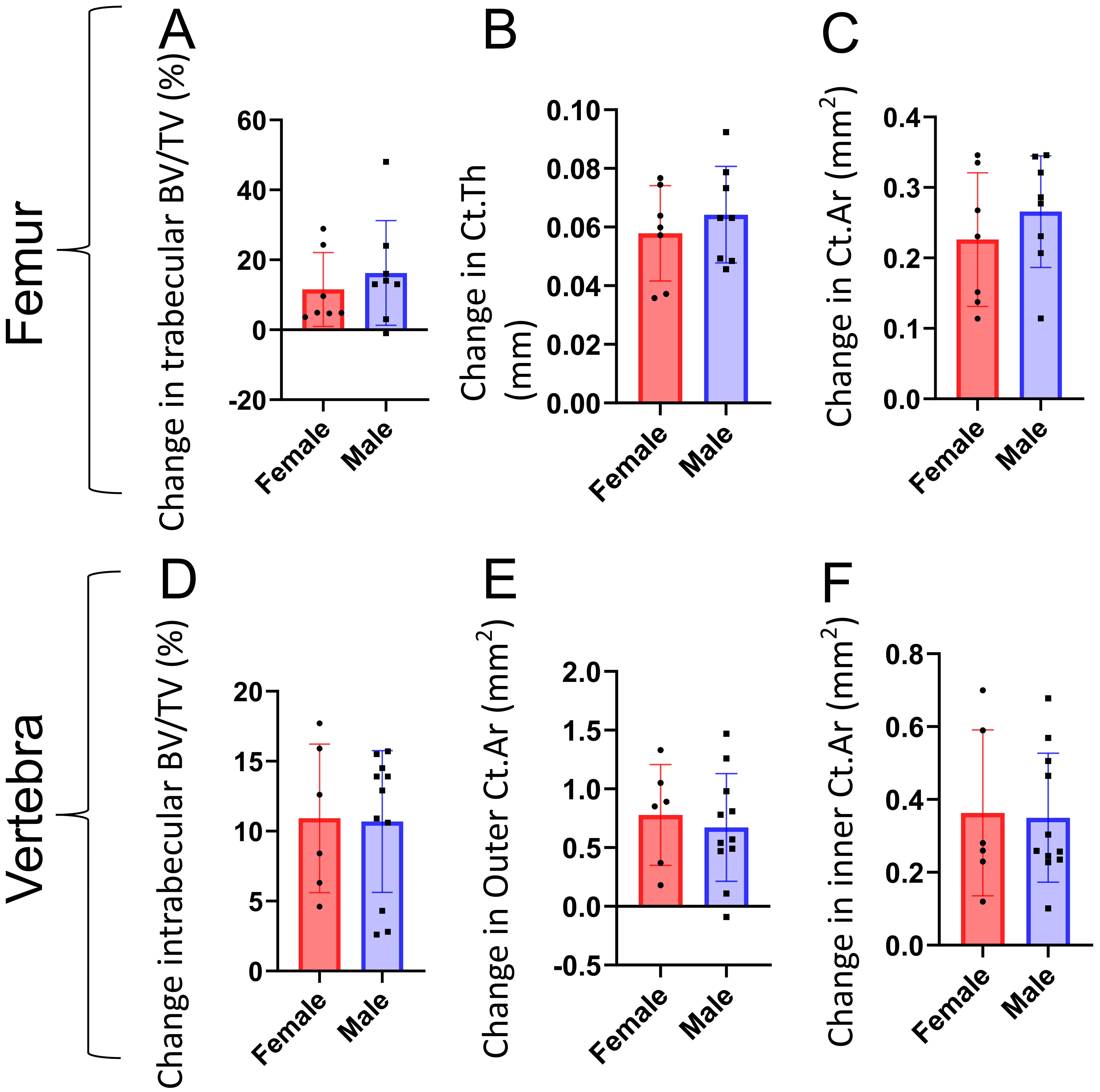

Figure S8. Female and male CKO mice have similar elevation in bone mass.

Changes in femoral (A-C) and vertebral (D-F) bone parameters in CKO mice compared to control (Chet) mice in respective sexes were calculated as: CKO values minus Chet values using the same data sets presented in Figs 4, 5, S3, S4. Each dot represents one mouse. Data are mean ± SD.

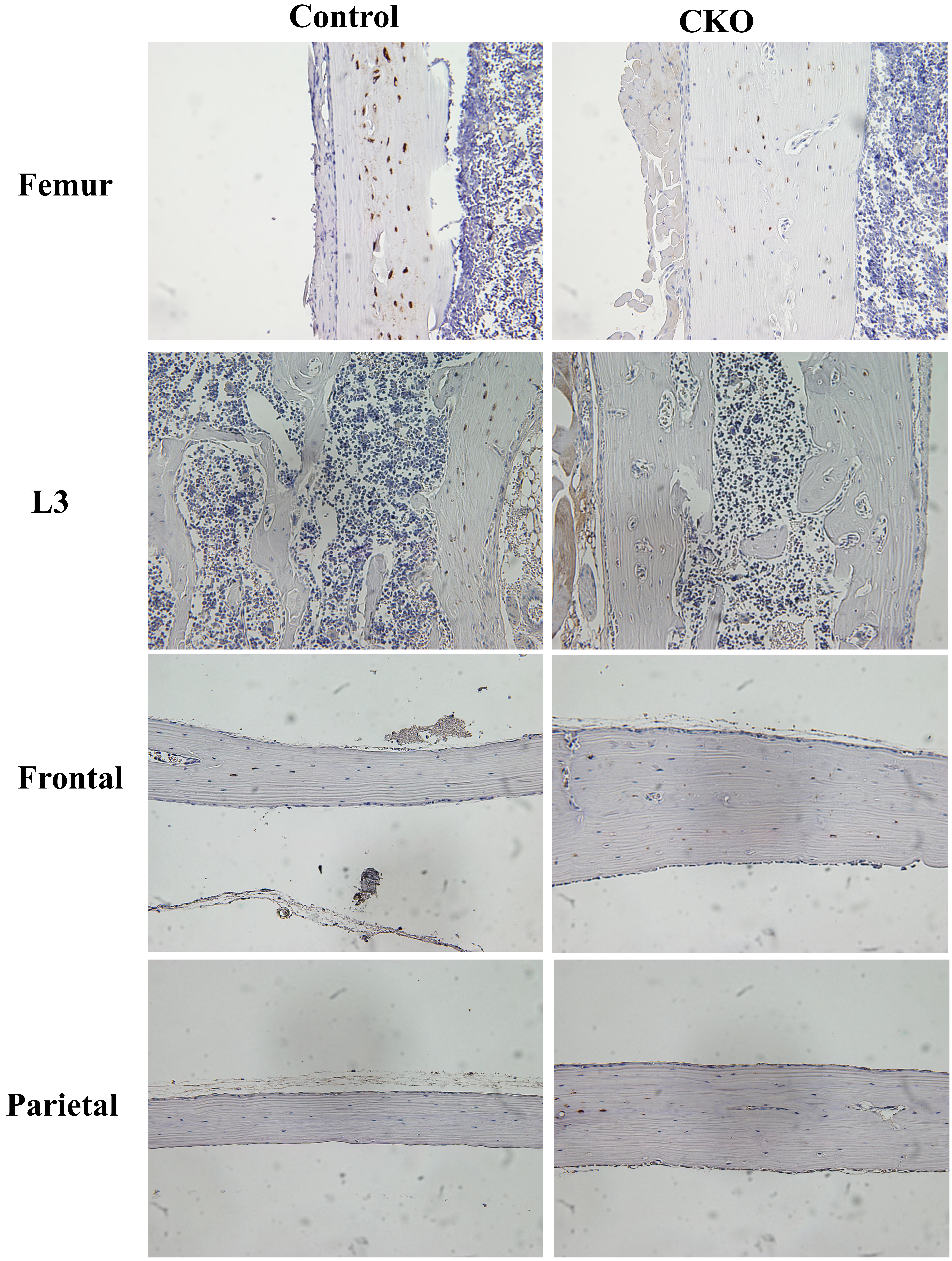

Fig. S9. Sclerostin expression in multiple skeletal sites following *Tsc1* deletion. Immunohistochemical staining for sclerostin in femur, L3 vertebra, frontal bone, and parietal bone from 2-month-old female control and CKO mice. In the femoral cortical bone, control osteocytes display strong sclerostin immunoreactivity, which is markedly reduced in CKO mice. In contrast, sclerostin staining in L3 vertebra, frontal bone, and parietal bone is generally low in control samples under the same staining conditions used for femur. Due to the low baseline signal in these skeletal sites, differences between control and CKO mice are less apparent.
